# Adsorption of Chlormethine Anti-Cancer Drug on Pure and Aluminum-Doped Boron Nitride Nanocarriers: A Comparative DFT Study

**DOI:** 10.3390/ph15101181

**Published:** 2022-09-23

**Authors:** Mahmoud A. A. Ibrahim, Al-shimaa S. M. Rady, Asmaa M. A. Mandarawe, Lamiaa A. Mohamed, Ahmed M. Shawky, Tamer H. A. Hasanin, Peter A. Sidhom, Mahmoud E. S. Soliman, Nayra A. M. Moussa

**Affiliations:** 1 Computational Chemistry Laboratory, Chemistry Department, Faculty of Science, Minia University, Minia 61519, Egypt; 2 School of Health Sciences, University of Kwa-Zulu-Natal, Westville, Durban 4000, South Africa; 3 Science and Technology Unit (STU), Umm Al-Qura University, Makkah 21955, Saudi Arabia; 4 Department of Chemistry, College of Science, Jouf University, Sakaka P.O. Box 2014, Saudi Arabia; 5 Department of Pharmaceutical Chemistry, Faculty of Pharmacy, Tanta University, Tanta 31527, Egypt; 6 Molecular Bio-Computation and Drug Design Research Laboratory, School of Health Sciences, University of Kwa-Zulu-Natal, Westville, Durban 4000, South Africa

**Keywords:** boron nitride nanocarriers, Chlormethine, anti-cancer drug, DFT calculations, thermodynamic parameters

## Abstract

The efficacy of pure and aluminum (Al)-doped boron nitride nanocarriers (B_12_N_12_ and AlB_11_N_12_) in adsorbing Chlormethine (CM), an anti-cancer drug, was comparatively dissected by means of the density functional theory method. The CM∙∙∙B_12_N_12_ and ∙∙∙AlB_11_N_12_ complexes were studied within two configurations, A and B, in which the adsorption process occurred via N∙∙∙ and Cl∙∙∙B/Al interactions, respectively. The electrostatic potential affirmations confirmed the opulent ability of the studied nanocarriers to engage in delivering CM via two prominent electrophilic sites (B and Al). Furthermore, the adsorption process within the CM∙∙∙AlB_11_N_12_ complexes was noticed to be more favorable compared to that within the CM∙∙∙B_12_N_12_ analog and showed interaction and adsorption energy values up to –59.68 and −52.40 kcal/mol, respectively, for configuration A. Symmetry-adapted perturbation theory results indicated that electrostatic forces were dominant in the adsorption process. Notably, the adsorption of CM over B_12_N_12_ and AlB_11_N_12_ nanocarriers exhibited predominant changes in their electronic properties. An elemental alteration was also revealed for the softness and hardness of B_12_N_12_ and AlB_11_N_12_ nanocarriers before and following the CM adsorption. Spontaneity and exothermic nature were obviously observed for the studied complexes and confirmed by the negative values of thermodynamic parameters. In line with energetic manifestation, Gibbs free energy and enthalpy change were drastically increased by the Al doping process, with values raised to –37.15 and –50.14 kcal/mol, respectively, for configuration A of the CM∙∙∙AlB_11_N_12_ complex. Conspicuous enhancement was noticed for the adsorption process in the water phase more than that in the gas phase and confirmed by the negative values of the solvation energy up to −53.50 kcal/mol for configuration A of the CM∙∙∙AlB_11_N_12_ complex. The obtained outcomes would be the linchpin for the future utilization of boron nitride as a nanocarrier.

## 1. Introduction

Owing to the tremendous blossoming of nanoscience in recent years, nanomaterials have been considered as potential candidates in a large array of applications such as optical devices [1], catalysis [2], and sensing materials [3]. Among the growing applications of nanomaterials, nanocarriers are of keen interest given their pivotal importance in the drug delivery process [4,5]. In the extending field of nanocarriers, fullerene-like structures were proved as potent drug nanocarriers, inspired by high biocompatibility and low cytotoxicity nature [6,7,8]. In the literature, group III nitride nanocarriers were found with exceptional properties, including elevated chemical stability, favorable band gap, suitable dielectric constant, considerable thermal conductivity, and upright oxidation resistance [9,10].

Furthermore, different geometries of boron nitrides (B_x_N_x_, where x refers to the number of boron and nitrogen atoms) were studied by Toftlund and Jensen, and the high stability of the B_12_N_12_ nanocarrier was addressed [11]. Soltani et al. confirmed that B_12_N_12_ was more preferable than B_16_N_16_ in the drug delivery process of a 5-aminolevulinic acid (5-AVA) drug [12]. By an arc-melting method, B_12_N_12_ was first synthesized with a band gap of 5.1 eV [13,14]. Afterward, B_12_N_12_ was extensively investigated to serve as a nanocarrier of diverse anti-cancer drugs such as Isoniazid [6], Amphetamine [15], Lomustine [16], etc. Consequently, the optical, electronic, and chemical features of the boron nitride (B_12_N_12_) nanocarrier were thoroughly under study. It was reported that a significant alteration in boron nitride features occurred through doping the B_12_N_12_ surface [17]. Concurrently, the aluminum (Al) atom is considered one of the most appropriate doping candidates due to its high porosity and ample surface active sites [18]. In fact, Al-doped boron nitride (AlB_11_N_12_) was reported as an excellent nanocarrier for several drugs, owing to the sharp changes in the band gap and the reversibility of the adsorption process [19]. In parallel, the AlB_11_N_12_ nanocarrier had a massive increment in its adsorption tendency, which was previously interpreted as an advantage of the Al doping processs [20,21]. Moreover, the adsorption processes of 5-aminosalicylic acid and the Cisplatin anti-cancer drug over the exterior surface of pristine and Al-doped B_12_N_12_ nanocarriers were investigated based on DFT calculations [22,23].

Chlormethine (CM), an anti-cancer drug with C_5_H_11_Cl_2_N chemical formula, was synthesized in 1935 and applied for cancer treatment in 1946. Pharmacologists proved the effectiveness of CM to treat prostate cancer, polycythemia vera, chronic myelocytic leukemia, and lymphosarcoma after clinical trials. Moreover, the topical formulation of CM was documented to be operative for different conditions such as skin diseases and cell lymphoma [24,25,26]. Nevertheless, CM had severe adverse effects that would eventually develop tentative blindness and skin cancer. With the aim of reducing undesirable side effects, versatile studies were conducted for using nanocarriers in the delivery process of CM. Subsequently, the adsorption process of CM over C_24_, B_12_C_6_N_6_, and B_12_N_12_ nanocarriers was investigated, revealing the crucial favorability for the B_12_N_12_ nanocarrier [27]. Moreover, the effect of transition metal-doped boron nitride was theoretically studied over the surfaces of the B_12_N_12_, ZnB_12_N_12_, NiB_12_N_12_, CoB_12_N_12_, FeB_12_N_12_, and CuB_12_N_12_ nanocarriers [28].

**Figure 1:**
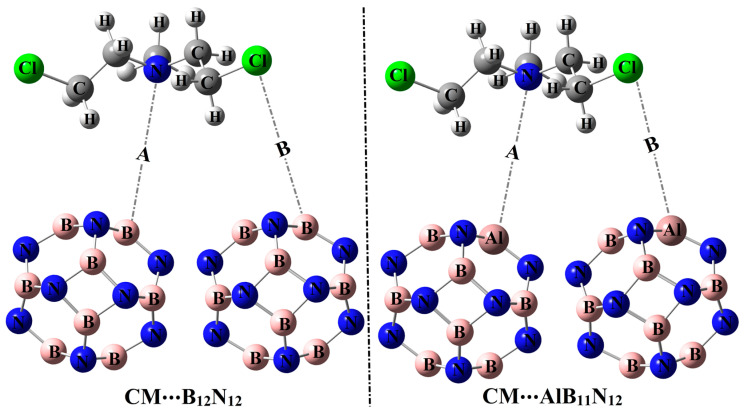
Pictorial representation for the adsorption process of the Chlormethine (CM) anti-cancer drug over pure and aluminum (Al)-doped boron nitride (B_12_N_12_ and AlB_11_N_12_) nanocarriers through configurations A and B.

In the current investigation, the predilection of pure and Al-doped boron nitride nanocarriers (B_12_N_12_ and AlB_11_N_12_) to adsorb CM anti-cancer drug within configurations A and B was discussed (Figure 1). By means of density functional theory (DFT) calculations, the occurrences of the adsorption process within the CM∙∙∙B_12_N_12_ and ∙∙∙AlB_11_N_12_ complexes were detected in both gas and water phases. A series of thermodynamic parameters were also computed to elucidate the spontaneity and nature of the adsorption process. The obtained findings would be essential for the development of the applications of pure and doped boron nitride nanocarriers in the drug delivery process of anti-cancer drugs.

## 2. Results

### 2.1. Electrostatic Potential (ESP) Analysis

**Figure 2:**
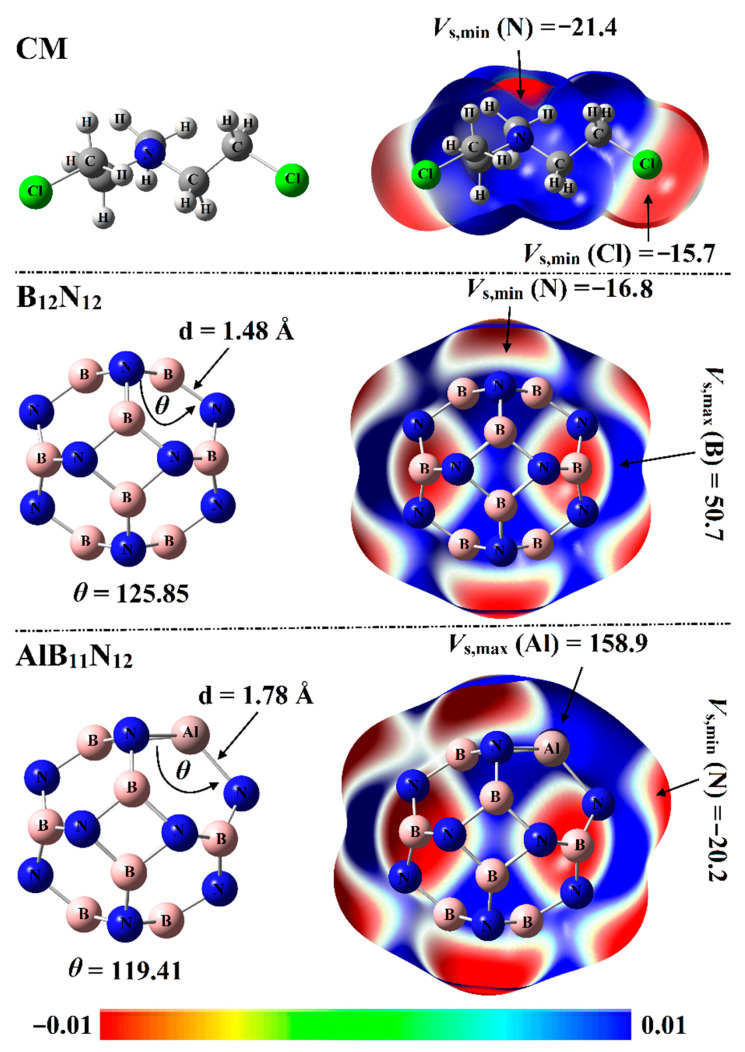
Optimized structures and molecular electrostatic potential (MEP) maps supplemented with electrostatic potential extrema (*V*_s,min_/*V*_s,max_, in kcal/mol) of CM, B_12_N_12_, and AlB_11_N_12_. The color scale changes from red (−0.01) to blue (+0.01) au.

Electrostatic potential (ESP) analysis has been introduced as a convenient and useful tool to qualitatively and quantitatively identify chemical systems [29]. For the optimized systems, molecular electrostatic potential (MEP) maps were generated using 0.002 au electron density envelopes according to the previous recommendations [30]. From a numerical point of view, surface electrostatic potential extrema were calculated in terms of maximum (*V*_s,max_) and minimum (*V*_s,min_) electrostatic potential values. The optimized structures with corresponding MEP maps of Chlormethine (CM) and pure and aluminum (Al)-doped boron nitride nanocarriers (B_12_N_12_ and AlB_11_N_12_) are graphed in Figure 2.

As delineated in Figure 2, the B_12_N_12_ nanocarrier with *T*_h_ symmetry was incorporated of identical eight hexagonal rings and six tetragonal rings. In terms of bond length, two nonequivalent B-N bond lengths were observed for the B_12_N_12_ nanocarrier: one between two hexagonal rings and the other one between a hexagonal and tetragonal ring. The B-N bond length was observed at 1.44 Å between two hexagonal rings and 1.48 Å between a hexagonal and tetragonal ring. Interestingly, the obtained bond length values were concomitant with previous studies [31,32].

For the AlB_11_N_12_ nanocarrier with the *C_s_* point group, the Al-N bond between two hexagonal rings was noticed with a length of 1.78 Å, while the Al-N bond between tetragonal and hexagonal was 1.82 Å. The bond length values illustrated in Figure 2 were found to be compatible with previous studies pertinent to the influence of Al doping on the configuration of the B_12_N_12_ nanocarrier [22].

From MEP maps visualized in Figure 2, two nucleophilic sites labeled with red regions were noticed along the surface of the CM drug, numerically ensured by *V*_s,min_ values of –15.7 and –21.4 kcal/mol along the surface of the Cl and N atoms, respectively. Turning to nanocarriers, positive ESP regions were observed over B and Al atoms, indicating the electrophilic nature of nanocarriers (Figure 2). Illustratively, *V*_s,max_ values over B and Al atoms of B_12_N_12_ and AlB_11_N_12_ nanocarriers were 50.7 and 158.9 kcal/mol, respectively. Evidently, the negative ESP on the N atoms in the AlB_11_N_12_ system was found with a higher *V*_s,min_ value compared with that in the B_12_N_12_ system, ensuring the prominent electron donation ability of the Al atom more than the B atom. Quantitatively, *V*_s,min_ values of N atoms were –16.8 and –20.2 kcal/mol for the B_12_N_12_ and AlB_11_N_12_ nanocarriers, respectively.

### 2.2. Adsorption Process

**Figure 3:**
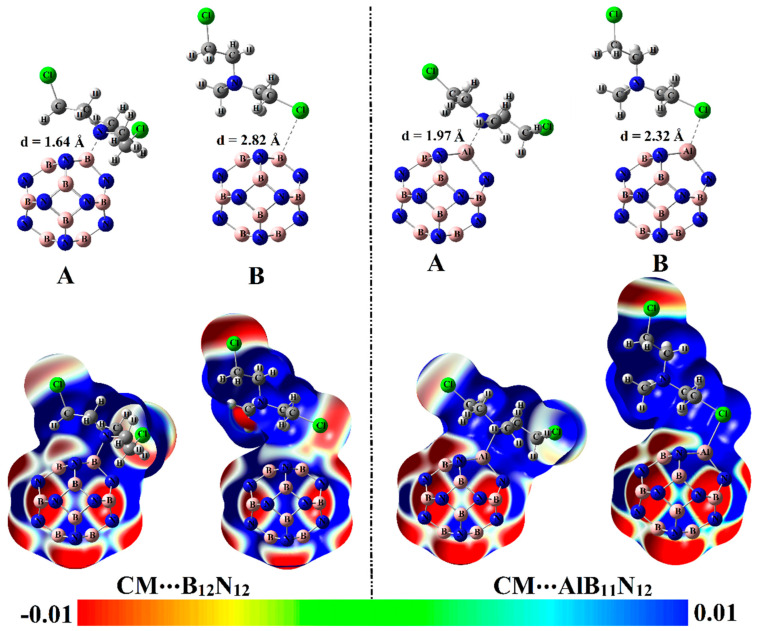
Optimized structures of CM∙∙∙B_12_N_12_ and ∙∙∙AlB_11_N_12_ optimized complexes within configurations A and B (at M06-2X/6-311+G** level of theory) along with molecular electrostatic potential (MEP) maps. MEP maps are plotted using electron density envelopes of 0.002 au. The color scale varies from −0.01 (red) to +0.01 (blue) au. Intermolecular distances (d) within the optimized complexes are given in Å.

The potentiality of pure and Al-doped boron nitride (B_12_N_12_ and AlB_11_N_12_) nanocarriers to adsorb the Chlormethine (CM) anti-cancer drug was thoroughly studied within configurations A and B for CM∙∙∙B_12_N_12_ and ∙∙∙AlB_11_N_12_ complexes (Figure 1). Geometrical optimization and MEP maps calculations were executed for the studied CM∙∙∙B_12_N_12_ and ∙∙∙AlB_11_N_12_ complexes, and the obtained structures are depicted in Figure 3. Table 1 compiles the interaction and adsorption energies of the optimized complexes along with intermolecular distances between the interacted species.

From the data listed in Table 1, substantial negative adsorption (*E*_ads_) and interaction (*E*_int_) energies of the CM∙∙∙B_12_N_12_ and ∙∙∙AlB_11_N_12_ complexes were observed, ensuring the occurrence of the adsorption process within modeled configurations. Notably, short CM∙∙∙B/∙∙∙Al intermolecular distances within the CM∙∙∙B_12_N_12_ and ∙∙∙AlB_11_N_12_ complexes were detected, with values ranging from 1.64 Å to 2.82 Å (Figure 3 and Table 1).

Among the studied complexes, the CM∙∙∙AlB_11_N_12_ complex had higher negative interaction and adsorption energies compared with CM∙∙∙B_12_N_12_ complex and showed values of –59.68 and –52.40 kcal/mol, respectively, within configuration A. The favorability of CM∙∙∙AlB_11_N_12_ complex could be ascribed to the further prominent electrophilic character of the AlB_11_N_12_ nanocarrier compared with the B_12_N_12_ analog in line with the *V*_s,max_ affirmations, which showed values of 158.9 and 50.7 kcal/mol, respectively. The interpretation of the superior adsorption behavior of the AlB_11_N_12_ nanocarrier toward the CM drug via site A more than B could be considered as a consequence of the higher negativity of ESP over the N atom compared with the Cl one that showed *V*_s,min_ values of –21.4 and –15.7 kcal/mol, respectively. The obtained results were quite similar in their foundations to the literature, which documented the highest adsorption favorability through the N site of the CM drug over X_3_O (X = Li, Na, and K)-doped B_12_N_12_ nanocarriers [33]. It is worth mentioning that the presented result of the CM∙∙∙B_12_N_12_ complex within configuration A is compatible with a previous study [27]. It was reported that the B_12_N_12_ nanocarrier showed preferable potency toward adsorbing the CM drug with *E*_ads_ up to −24.33 kcal/mol. The energy difference between the obtained and previously reported *E*_ads_ values could be mainly relevant to the variant in the utilized levels of computations.

### 2.3. SAPT Calculations

**Figure 4:**
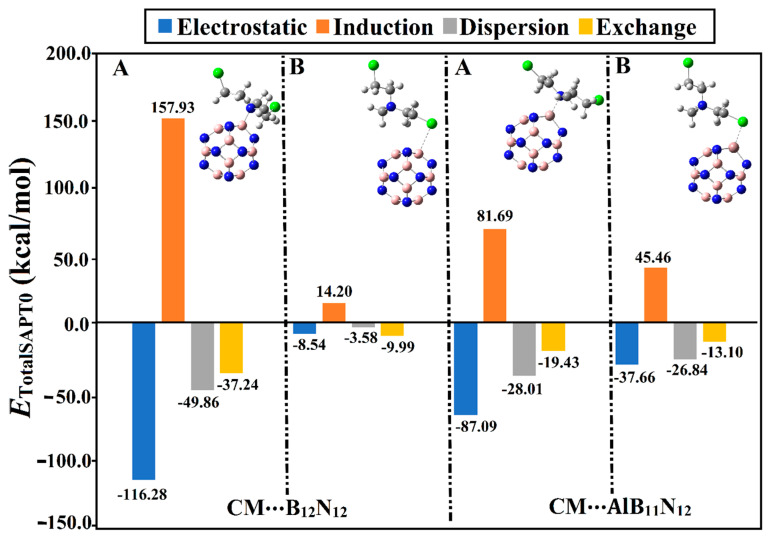
Bar chart presenting the physical components (i.e., electrostatic (*E*_elst_), induction (*E*_ind_), dispersion (*E*_disp_), and exchange (*E*_exch_)) of the total SAPT0 energies for the CM∙∙∙B_12_N_12_ and ∙∙∙AlB_11_N_12_ optimized complexes in configurations A and B.

Symmetry-adapted perturbation theory (SAPT) analysis was conducted to decompose the interaction energy into its four main physical forces, namely, electrostatic (*E*_elst_), exchange (*E*_exch_), dispersion (*E*_disp_), and induction (*E*_ind_) forces. For the optimized complexes, SAPT analysis was performed at the SAPT0 level of theory applying PSI4 code [34]. The four physical components of the interaction energy of Chlormethine with B_12_N_12_ and AlB_11_N_12_ nanocarriers are graphically correlated in Figure 4.

It can be observed from Figure 4 that the electrostatic forces (*E*_elst_) governed the adsorption process within the CM∙∙∙B_12_N_12_ and ∙∙∙AlB_11_N_12_ complexes. Notably, the dispersion (*E*_dis_) and induction (*E*_ind_) forces exhibited preferable contributions, reinforcing the occurrence of the adsorption process. On the other hand, exchange forces (*E*_exch_) were found with positive values, demonstrating their unfavorable contribution to the forces beyond the adsorption process. Illustratively, the energetic values of *E*_elst_, *E*_ind_, *E*_disp_, and *E*_exch_ were found to be –87.09, –28.01, –19.43, and 81.69 kcal/mol, respectively, for the CM∙∙∙AlB_11_N_12_ complex within configuration A. Apparently, the obtained total SAPT0 energies were observed to be in line with the adsorption and interaction energy values (Table 1). Numerically, the total SAPT0 energies of the CM∙∙∙B_12_N_12_ and ∙∙∙AlB_11_N_12_ complexes within configurations A/B were –45.46/–7.91 and –59.74/–32.74 kcal/mol, respectively.

Although the *E*_elst_ and *E*_exch_ components of the CM∙∙∙B_12_N_12_ complex within configuration A showed higher values compared with that of the CM∙∙∙AlB_11_N_12_ complex, the total SAPT0 energy of the latter was higher than the former. Although the total SAPT0 energy of the CM∙∙∙AlB_11_N_12_ complex within configuration A was higher than that of the CM∙∙∙B_12_N_12_ analog, the *E*_elst_ and *E*_exch_ components of the latter complex showed higher values compared with those of the former.

Although the *E*_elst_ components of the CM∙∙∙B_12_N_12_ complex within configuration A showed a higher negative value compared with that of the CM∙∙∙AlB_11_N_12_ complex, the total SAPT0 energy of the latter complex was higher than the former. This observation could be interpreted by considering the *E*_exch_ component, which exhibited a higher positive value for the latter complex than the former. Illustratively, *E*_elst_ values were –116.28 and –87.09 kcal/mol for the CM∙∙∙B_12_N_12_ and ∙∙∙AlB_11_N_12_ complexes, respectively, within configuration A. In contrast, *E*_exch_ values for CM∙∙∙B_12_N_12_ and ∙∙∙AlB_11_N_12_ complexes within configuration A were found to be 157.93 and 81.69 kcal/mol, respectively.

### 2.4. QTAIM and NCI Calculations

**Figure 5:**
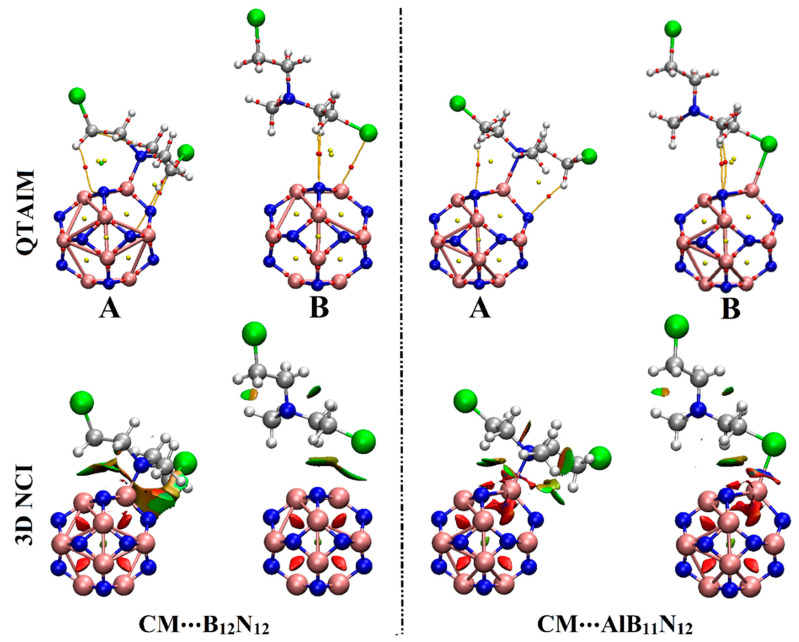
Visualized QTAIM and 3D NCI diagrams of the optimized CM∙∙∙B_12_N_12_ and ∙∙∙AlB_11_N_12_ complexes in configurations A and B. In QTAIM diagrams, red dots represent the location of BCPs and BPs. Three-dimensional NCI isosurfaces are graphed with a reduced density gradient value of 0.50 au and colored from blue to red according to sign (λ_2_)*ρ* ranging from −rangi (blue) to 0.020 (red) au.

Quantum theory of atoms in molecules (QTAIM) and noncovalent interaction (NCI) index analyses were considered as informative investigations for visualizing inter- and intra-molecular interactions [35]. Upon QTAIM and NCI, the adsorption of a drug over nanocarriers was endorsed by electron density and its derivatives. By applying QTAIM analysis, bond paths (BPs) and bond critical points (BCPs) were generated. The topological derivatives, inclusive total energy density (H_b_), Laplacian (∇^2^*ρ*_b_), electron density (*ρ*_b_), kinetic electron density (G_b_), local potential electron energy density (V_b_), and negative ratio of kinetic and potential electron energy density (−G_b_/V_b_) were calculated and are gathered in Table 2. Three-dimensional isosurface graphs were generated using sign (λ_2_)*ρ* with colors ranging from blue (–0.035 au) to red (0.020 au). QTAIM and 3D NCI diagrams of the CM∙∙∙B_12_N_12_ and ∙∙∙AlB_11_N_12_ optimized complexes are depicted in Figure 5.

As explicitly shown in Figure 5, the tendency of B_12_N_12_ and AlB_11_N_12_ nanocarriers to adsorb the CM drug was clearly confirmed by the visualized bond critical points (BCPs) and bond paths (BPs) between the interacted species (i.e., N/Cl of CM drug and B/Al of B_12_N_12_/AlB_11_N_12_ nanocarriers). Evidently, the contributions of secondary interactions that supported the adsorption process within the studied complexes were noticed via the existence of variant BCPs and BPs between the hydrogen atom of the CM drug and the N atom of B_12_N_12_/AlB_11_N_12_ nanocarriers (Figure 5).

According to the data in Table 2, the partial covalent and electrostatic nature of the adsorption process within the CM∙∙∙B_12_N_12_ and ∙∙∙AlB_11_N_12_ complexes were generally unveiled. The obtained negative H_b_ values, positive ∇^2^*ρ*_b_ values, and values less than the unity of –G_b_/V_b_ outlined the partial covalent and electrostatic nature of the interactions within the CM∙∙∙B_12_N_12_ (configuration A) and ∙∙∙AlB_11_N_12_ (configurations A and B) complexes [36]. For instance, H_b_, ∇^2^*ρ*_b_, and –G_b_/V_b_ of the CM∙∙∙AlB_11_N_12_ complex within configuration A were –0.0046, 0.3581, and 0.9530 au, respectively. The CM∙∙∙B_12_N_12_ within configuration B was noticed with a weak electrostatic nature that was detected by the positive H_b_ values, positive ∇^2^*ρ*_b_ values, and values exceeding the unity for –G_b_/V_b_. Numerically, H_b_, ∇^2^*ρ*_b_, and –G_b_/V_b_ of the CM∙∙∙B_12_N_12_ complex within configuration B were 0.0007, 0.0387, and 1.0842 au, respectively.

On 3D NCI isosurfaces, the tendency of the B_12_N_12_ and AlB_11_N_12_ nanocarriers to adsorb the CM dug was elucidated by the occurrence of colored isosurfaces between the interacted species within the studied complexes. Moreover, the change in the color scale of the 3D NCI isosurfaces was found to be concurrent with the adsorption energy pattern; the strongest and weakest interactions were observed in blue and green colors, respectively.

### 2.5. Electronic Parameters

**Figure 6:**
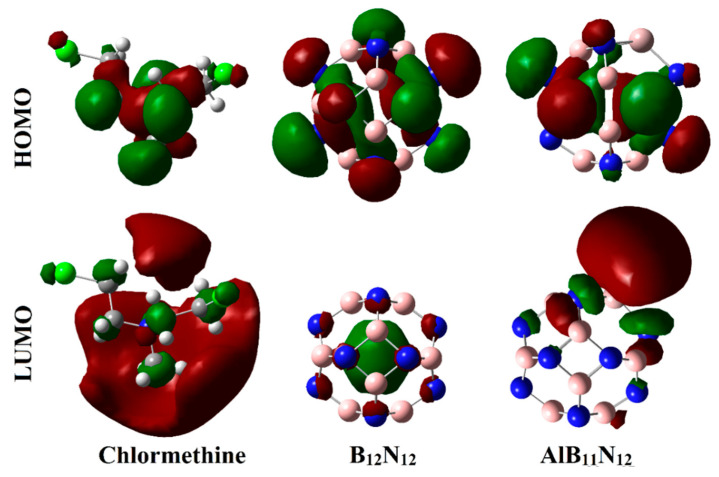
Diagrams of HOMO and LUMO distributions of CM, B_12_N_12_, and AlB_11_N_12_ systems.

**Figure 7:**
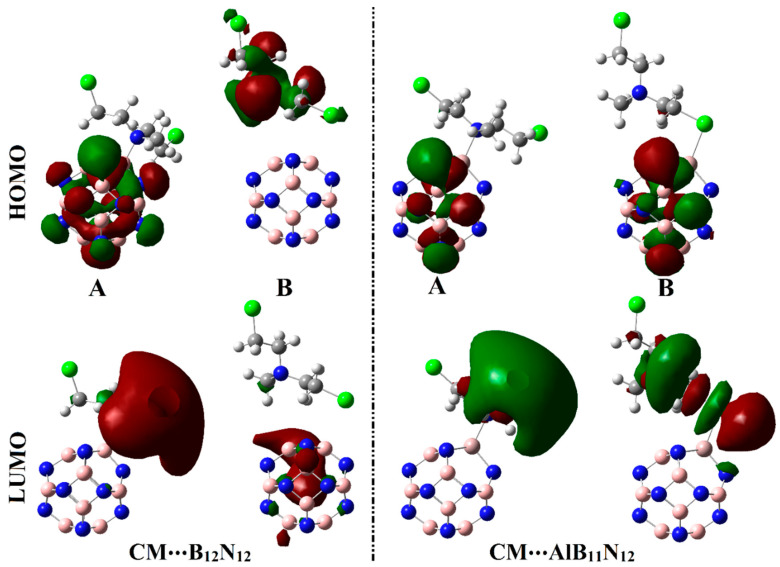
Diagrams of HOMO and LUMO distributions of the optimized CM∙∙∙B_12_N_12_ and ∙∙∙AlB_11_N_12_ complexes within configurations A and B.

The impact of the adsorption process of Chlormethine (CM) over pure and Al-doped boron nitride (B_12_N_12_ and AlB_11_N_12_) nanocarriers was investigated by elucidating their densities and electronic energy levels. To this end, the highest occupied molecular orbital (*E*_HOMO_), Fermi level (*E*_FL_), and the lowest unoccupied molecular orbital (*E*_LUMO_), were calculated before and after the adsorption process. Subsequently, the energy gap (*E*_gap_) was computed as the difference between the *E*_LUMO_ and E_HOMO_. Figure 6 and Figure 7 show the electron densities of HOMO/LUMO for the isolated systems and complexes, respectively. The obtained *E*_HOMO_, E_FL_, *E*_LUMO_, and *E*_gap_ values are given in Table 3.

As exemplified in Figure 6, the distributions of HOMO and LUMO represented the densities purely around the nucleophilic and electrophilic sites, respectively. Apparently, HOMO distributions were concentrated around the N and Cl atoms of the CM, while the LUMO ones were denoted over B and Al atoms of B_12_N_12_ and AlB_11_N_12_, respectively. Upon contact with CM, obvious changes in HOMO and LUMO distributions were identified, as is apparent in Figure 7. HOMO and LUMO levels were found over nanocarrier and drug, respectively, within the studied complexes, with an exception for the CM∙∙∙B_12_N_12_ complex within configuration B. This observation outlined that the occurrence of the adsorption process is attributed to the charge transfer between drug and nanocarriers in all the studied complexes. The HOMO and LUMO affirmations were consistent with the energetic ones summarized in Table 1, which highlighted the lesser favorability of the CM∙∙∙B_12_N_12_ complex within configuration B compared with other studied complexes.

According to the data in Table 3, the *E*_HOMO_, *E*_FL_, *E*_LUMO_, and *E*_gap_ values of the pure B_12_N_12_ nanocarrier were evidently changed after doping by an Al atom in the AlB_11_N_12_ nanocarrier. From Table 3, the *E*_HOMO_, *E*_FL_, *E*_LUMO_, and *E*_gap_ values of B_12_N_12_/AlB_11_N_12_ nanocarriers were –9.54/–9.10, –5.12/–7.05, –0.35/–2.50, and 9.19/6.60 eV, respectively. These numerical values particularly addressed the contributions of Al doping in destabilizing and stabilizing *E*_HOMO_ and *E*_LUMO_ of the pure B_12_N_12_ nanocarrier.

It is also worth mentioning that an obvious variation in *E*_HOMO_ and *E*_LUMO_ values was unveiled after the adsorption process of the CM drug over the B_12_N_12_ and AlB_11_N_12_ nanocarriers. Notably, a slight decline in *E*_HOMO_ and *E*_FL_ values was found after the adsorption process. For instance, *E*_HOMO_ of B_12_N_12_ nanocarrier was –9.54 eV, and it diminished to –8.92 and –8.30 eV for CM∙∙∙B_12_N_12_ complexes within configurations A and B, respectively. Generally, obvious downshifts and upshifts in the *E*_LUMO_/*E*_gap_ values were observed after the adsorption process within CM∙∙∙B_12_N_12_ and ∙∙∙AlB_11_N_12_ complexes, respectively.

### 2.6. Global Indices of Reactivity

Global indices of reactivity were computed before and after the adsorption process to comprehensively clarify the impact of adsorption of the CM drug over B_12_N_12_ and AlB_11_N_12_ nanocarriers. Various parameters, including ionization potential (*IP*), electron affinity (*EA*), chemical potential (*μ*), global hardness (*η*), global softness (*S*), electrophilicity index (*ω*), and work function (*Φ*), were calculated and are compiled in Table 4.

With regard to global indices of reactivity, a significant difference was denoted in the values of *IP*, *EA*, *μ*, *η*, *S*, *ω*, and *Φ* between the pure and Al-doped boron nitride (B_12_N_12_ and AlB_11_N_12_) nanocarriers. From summarized data in Table 4, the *IP*, *EA*, *μ, η*, *S*, *ω*, and *Φ* values of B_12_N_12_/AlB_11_N_12_ were 9.54/9.10 eV, 0.35/2.50 eV, –4.94/–5.80 eV, 4.60/3.30 eV, 0.22/0.30 eV^–1^, 2.66/5.10 eV, and 5.12/7.05 eV, respectively. Consequently, the Al doping enhanced the properties of the pure boron nitride nanocarrier to be a suitable surface for adsorbing the CM drug by declining and increasing the values of *IP*/*η* and *EA*/*μ*/*S*/*ω*/*Φ*, respectively.

Moreover, Table 4 offers insight into the substantial effect of the adsorption process of the CM drug over pure and Al-doped boron nitride (B_12_N_12_ and AlB_11_N_12_) nanocarriers via sizeable changes in the calculated reactivity parameters. As detailed in Table 4, *IP* values of the B_12_N_12_ and AlB_11_N_12_ nanocarriers decreased upon contact with the CM drug from 9.54 and 9.10 eV to 8.92 and 8.55 eV, respectively, within configuration A. The same pattern was noticed in the case of *μ, ω*, and *Φ*, and an irregular pattern was noticed for *EA*, *η*, and *S* values, which could be ascribed to the main dependence of these values on *E*_LUMO_. Overall, the noticeable difference in the global indices of reactivity parameters extensively ensured the adequacy of the B_12_N_12_ and AlB_11_N_12_ nanocarriers for adsorbing the CM drug, along with highlighting the preferential role of the Al doping process.

### 2.7. DOS Analysis

**Figure 8:**
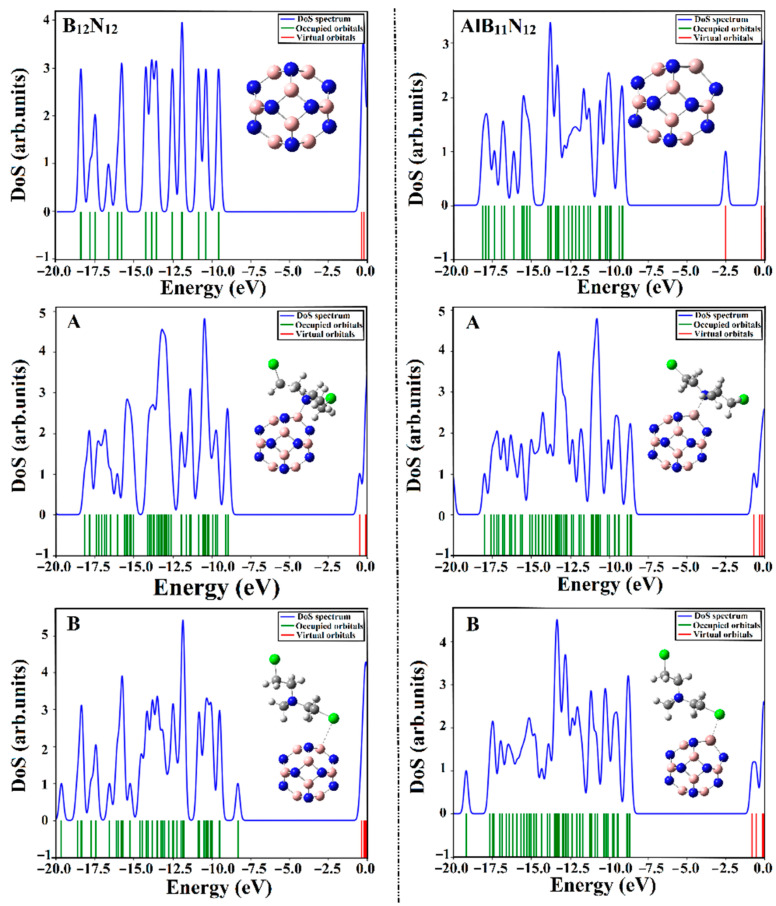
Density of states (DOS) plots for the B_12_N_12_ and AlB_11_N_12_ nanocarriers before and after the adsorption process of CM within the CM∙∙∙B_12_N_12_ and ∙∙∙AlB_11_N_12_ complexes within configurations A and B.

The density of states (DOS) analysis was apprehended for B_12_N_12_ and AlB_11_N_12_ nanocarriers before and after the adsorption process of the CM drug over B_12_N_12_ and AlB_11_N_12_ nanocarriers. DOS diagrams were generated and are expressed in Figure 8.

As is evident in Figure 8, the energy states within the DOS diagram of the AlB_11_N_12_ nanocarrier were found to be more concentrated than the B_12_N_12_ analog, revealing the favorable impact of the Al doping process on the electronic properties of the pure boron nitride (B_12_N_12_) nanocarrier.

It is apparent that the occurrence of the adsorption process within the CM∙∙∙B_12_N_12_ and ∙∙∙AlB_11_N_12_ complexes was thoroughly confirmed by the appearance of new energy states around the Fermi level in the extracted DOS diagrams. The resulted states were noticed more obviously in the case of CM∙∙∙AlB_11_N_12_ complexes compared with CM∙∙∙B_12_N_12_, in line with energetic patterns. Indeed, an obvious decrement in *E*_gap_ was noticed following the adsorption of CM that in turn enhanced the conductivity (Equation (17)) of the investigated nanocarriers. Overall, the obtained results affirmed that the B_12_N_12_ and AlB_11_N_12_ nanocarriers are promising electrochemical biosensors for the Chlormethine (CM) anti-cancer drug.

### 2.8. Solvent Effect

Toward tackling the influence of the solvent on the adsorption process of the CM drug over B_12_N_12_ and AlB_11_N_12_ nanocarriers, the polarizable continuum model was utilized for water as a solvent. For the CM∙∙∙B_12_N_12_ and ∙∙∙AlB_11_N_12_ complexes, the calculations of geometrical optimization were executed in the presence of water. Then, adsorption ($E_{\mathrm{ads}}^{\mathrm{solvent}}$) and solvation (Δ*E*_solv_) energies were computed for the obtained optimized complexes and are given in Table 5.

According to summarized data in Table 5, negative values of $E_{\mathrm{ads}}^{\mathrm{solvent}}$ were noticed for all the considered complexes, clarifying the potential of the B_12_N_12_ and AlB_11_N_12_ nanocarriers to adsorb CM in the water phase. In line with the data in Table 1, the energetics of the studied complexes in the water phase within configuration A were more preferable compared to B. For example, $E_{\mathrm{ads}}^{\mathrm{solvent}}$ values of the CM∙∙∙B_12_N_12_ complex within configurations A and B were –30.86 and –4.06 kcal/mol, respectively.

Furthermore, the CM∙∙∙AlB_11_N_12_ complexes were noticed with higher negative values of $E_{\mathrm{ads}}^{\mathrm{solvent}\;}$ compared with CM∙∙∙B_12_N_12_ analogs. This observation consistently ensured the favorable Al doping contributions to the adsorption process in the water phase, as Table 5 shows. For instance, the CM∙∙∙B_12_N_12_ and ∙∙∙AlB_11_N_12_ complexes within configuration A exhibited $E_{\mathrm{ads}}^{\mathrm{solvent}}$ with values of –30.86 and –51.30 kcal/mol, respectively. Further favorability of the occurrence of the adsorption process was affirmed in the water phase more than in the gas phase and confirmed by the negative values of the Δ*E*_solv_.

### 2.9. Thermodynamic Parameters

To deeply understand the nature of the adsorption process of the CM drug on the B_12_N_12_ and AlB_11_N_12_ nanocarriers, the thermodynamic parameters were computed. The thermodynamic parameters for CM∙∙∙B_12_N_12_ and ∙∙∙AlB_11_N_12_ optimized complexes, at 1 atm and 298 K, Gibbs free energy (Δ*G*), enthalpy change (Δ*H*), and entropy change (Δ*S*) were calculated and are given in Table 6.

As shown in Table 6, the spontaneity of the adsorption process within the CM∙∙∙B_12_N_12_ and ∙∙∙AlB_11_N_12_ complexes was confirmed via the negative values of Δ*G*. Furthermore, negative Δ*H* values were an indication of the exothermic nature of the adsorption process within all considered complexes. Small and neglectable values of Δ*S* were noticed for all the studied complexes. Similar to the adsorption energy pattern, higher negative values of thermodynamic energetic quantities were mainly pertinent to the CM∙∙∙AlB_11_N_12_ complexes, compared with CM∙∙∙B_12_N_12_ complexes. From enrolled data in Table 6, the Δ*G*, Δ*H*, and Δ*S* of the CM∙∙∙AlB_11_N_12_ complexes within configuration A were –37.15, –50.14, and –0.04 kcal/mol, respectively.

### 2.10. Recovery Time

Recovery time (*τ*) is informative in the desorption process and is mainly correlated with the adsorption energy. For the CM∙∙∙B_12_N_12_ complexes within configurations A and B, *τ* values were 0.02 nS and 0.01 pS, respectively. In comparison, *τ* values of the CM∙∙∙AlB_11_N_12_ complexes within configurations A and B were 1.4 × 10^8^ S and 0.11 μS, respectively. Apparently, the CM∙∙∙B_12_N_12_ complex within configuration B, the least favorable energetic complex, had the shortest recovery time value, indicating its preferential feasibility in the desorption process. Contrarily, the most favorable CM∙∙∙AlB_11_N_12_ complex within configuration A had the longest recovery time, which is consistent with its substantial negative adsorption energy. Based on these results, the B_12_N_12_ and AlB_11_N_12_ nanocarriers would be preferential for adsorbing the CM drug within the modeled configurations given their short recovery time.

## 3. Computational Methods

The adsorption process of the Chlormethine (CM) anti-cancer drug over the exterior surface of pure and aluminum (Al)-doped boron nitride nanocarriers (B_12_N_12_ and AlB_11_N_12_) was comparatively scrutinized. The CM∙∙∙B_12_N_12_ and ∙∙∙AlB_11_N_12_ complexes were modeled within configurations A and B, in which the adsorption process occurred via N∙∙∙ and Cl∙∙∙B/Al interactions (see Figure 1). To follow the drug loading process, CM, B_12_N_12_, and AlB_11_N_12_ structures were fully optimized at Minnesota 2006 with the double Hartree–Fock exchange function (M06-2X) [37] along with the 6-311+G** basis set. Based on the optimized structures, electrostatic potential analysis (ESP) was conducted to clarify the electron-rich and -deficient sites over the molecular surface of the investigated systems. To acquire pictorial descriptions, molecular electrostatic potential (MEP) maps were created using electron density envelopes of 0.002 au [30]. Furthermore, using ESP, the numerical description was obtained by executing and extracting surface electrostatic potential extrema (*V*_s,min_/*V*_s,max_) with the help of Multiwfn 3.7 software [38].

Upon CM∙∙∙B_12_N_12_ and ∙∙∙AlB_11_N_12_ optimized complexes, adsorption (*E*_ads_) and interaction (*E*_int_) energies were calculated, and the counterpoise correction method was considered to abolish the basis set superposition error (BSSE) as follows:

(1) $$E_{\mathrm{ads}\;}=E_{\mathrm{CM}\cdot \cdot \cdot B_{12}N_{12}/\cdot \cdot \cdot {\mathrm{AlB}}_{11}N_{12}}-\left(E_{\mathrm{CM}}+E_{B_{12}N_{12}/{\mathrm{AlB}}_{11}N_{12}}\right)+E_{\mathrm{BSSE}}$$

(2) $$E_{\mathrm{int}\;}=E_{\mathrm{CM}\cdot \cdot \cdot B_{12}N_{12}/\cdot \cdot \cdot {\mathrm{AlB}}_{11}N_{12}}-\left(E_{\mathrm{CM}\;\mathrm{in}\;\mathrm{complex}}+E_{B_{12}N_{12}/{\mathrm{AlB}}_{11}N_{12}\mathrm{in}\;\mathrm{complex}}\right)+E_{\mathrm{BSSE}}$$

where $E_{\mathrm{CM}\cdot \cdot \cdot B_{12}N_{12}/\cdot \cdot \cdot {\mathrm{AlB}}_{11}N_{12}}$, $E_{\mathrm{CM}}$, and $E_{B_{12}N_{12}/{\mathrm{AlB}}_{11}N_{12}}$ represent energies of the complex, the isolated CM drug, and the isolated B_12_N_12_/AlB_11_N_12_ nanocarrier, respectively. $E_{\mathrm{CM}\;\mathrm{in}\;\mathrm{complex}}$ and $E_{B_{12}N_{12}/{\mathrm{AlB}}_{11}N_{12}\mathrm{in}\;\mathrm{complex}}$ are the energies of the CM drug and the B_12_N_12_/AlB_11_N_12_ nanocarrier based on their respective coordinates in the optimized CM∙∙∙B_12_N_12_/∙∙∙AlB_11_N_12_ complexes.

To unveil the physical forces that controlled the adsorption process, symmetry-adapted perturbation theory (SAPT) analysis was executed with the help of the PSI4 package [34]. Using SAPT analysis, total adsorption energies could be portioned into four physical components, namely, electrostatic (*E*_elst_), exchange (*E*_exch_), induction (*E*_ind_), and dispersion (*E*_disp_) forces. Within SAPT analysis, the SAPT0 level of truncation was applied, and Total SAPT0 energies were calculated as described in the following equations [39]:

(3) $$E^{\mathrm{SAPT}0}=E_{\mathrm{elst}}+E_{\mathrm{exch}}+E_{\mathrm{ind}}+E_{\mathrm{disp}}$$

where:

(4) $$E_{\mathrm{elst}}=E_{\mathrm{elst}}^{\left(10\right)}$$

(5) $$E_{\mathrm{exch}}=E_{\mathrm{exch}}^{\left(10\right)}$$

(6) $$E_{\mathrm{ind}}=E_{\mathrm{indresp}}^{\left(20\right)}+E_{\mathrm{exch}-\mathrm{indresp}}^{\left(20\right)}+\;\delta E_{\mathrm{HFresp}}^{\left(2\right)}$$

(7) $$E_{\mathrm{disp}}=E_{\mathrm{disp}}^{\left(20\right)}+E_{\mathrm{exch}-\mathrm{disp}}^{\left(20\right)}$$

The calculations of the quantum theory of atoms in molecules (QTAIM) in addition to the noncovalent interaction (NCI) index were also performed. Using QTAIM analysis, bond critical points (BCPs) and bond paths (BPs) were induced. Topological features, including total energy density (H_b_), Laplacian (∇^2^*ρ*_b_), electron density (*ρ*_b_), kinetic electron density (G_b_), local potential electron energy density (V_b_), and negative ratio of kinetic and potential electron energy density (−G_b_/V_b_), were also calculated. Three-dimensional NCI diagrams were generated and visualized using (λ_2_)*ρ* with values from –0.035 to 0.020 au and represented by colors ranging from blue to red, respectively. QTAIM and NCI analyses were achieved with the help of Multiwfn 3.7 [38] and pictorially represented using the Visual Molecular Dynamics (VMD) program [40].

To elucidate the electronic parameters, the frontier molecular orbitals (FMOs) theory was invoked. Within the context of FMOs, the electron density distribution of the highest occupied molecular orbital (HOMO) and the lowest unoccupied molecular orbital (LUMO) were plotted before and after adsorption. In addition, energies of HOMO (*E*_HOMO_) and LUMO (*E*_LUMO_) were computed. Based on *E*_HOMO_ and *E*_LUMO_ values, the Fermi level (*E*_FL_) and LUMO–HOMO energy gap (*E*_gap_) were calculated by utilizing Equations (8) and (9), respectively.

(8) $$E_{\mathrm{FL}}=E_{\mathrm{HOMO}}+\frac{E_{\mathrm{LUMO}}-E_{\mathrm{HOMO}}}{2}$$

(9) $$E_{\mathrm{gap}}=E_{\mathrm{LUMO}}-E_{\mathrm{HOMO}}$$

Various electronic properties, including ionization potential (*IP*), electron affinity (*EA*), chemical potential (*μ*), global hardness (*η*), global softness (*S*), electrophilicity index (*ω*), and work function (*Φ*), were computed for CM, B_12_N_12_, and AlB_11_N_12_ molecules before and after adsorption, as follows:

(10) $$IP=-E_{\mathrm{HOMO}}$$

(11) $$EA=-E_{\mathrm{LUMO}}$$

(12) $$\eta =\frac{E_{\mathrm{LUMO}}-E_{\mathrm{HOMO}}}{2}$$

(13) $$\mu =\frac{E_{\mathrm{LUMO}}+E_{\mathrm{HOMO}\;}}{2}$$

(14) $$s=\frac{1}{\eta }$$

(15) $$\omega =\frac{\mu^{2}}{2\eta }$$

(16) $$\Phi =V_{\mathrm{eL}\left(+\infty \right)}-E_{\mathrm{FL}}$$

where V_eL_(+∞) is the vacuum-level electrostatic potential that is assumed to be ≈ 0. In parallel, to provide further evidence of the attractive nature of pure and Al-doped (B_12_N_12_ and AlB_11_N_12_) nanocarriers toward CM adsorption, electrical conductivity was elucidated utilizing the following equation:

(17) $$\sigma \;\alpha \;\mathrm{Exp}\left(\frac{-E_{\mathrm{gap}}}{\mathrm{kT}}\right)$$

where σ refers to electrical conductivity, *E*_gap_ is the energy gap, k is Boltzmann’s constant, and T characterizes temperature. The density of states (DOS) plots were generated using GaussSum software [41]. To explicitly treat the effect of water as a solvent, the polarizable continuum model (PCM) method was applied [42,43]. For the optimized complexes, the solvation ($\Delta E_{\mathrm{solv}}$) energy was computed as the difference between the total energies in the water and gas phases according to Equation (18). The adsorption energies ($E_{\mathrm{ads}}^{\mathrm{solvent}})$ in the water phase were also assessed.

(18) $$\Delta E_{\mathrm{solv}}=E_{\mathrm{solvent}}-E_{gas}$$

For a more quantitative view of favorability, the thermodynamic parameters including changes in Gibbs free energy (Δ*G*), enthalpy (Δ*H*), and entropy (Δ*S*) were calculated for the studied complexes by applying frequency calculations [44]. The change in thermodynamic parameters was presented as the following equation:

(19) $$\Delta M=M_{\mathrm{CM}\cdot \cdot \cdot B_{12}N_{12}/\cdot \cdot \cdot {\mathrm{AlB}}_{11}N_{12}}-\left(M_{\mathrm{CM}}+M_{B_{12}N_{12}/{\mathrm{AlB}}_{11}N_{12}}\right)+E_{\mathrm{BSSE}}$$

where Δ*M* represents the quantity of Δ*G*, Δ*H*, and Δ*S*. Furthermore, $M_{\mathrm{CM}\cdot \cdot \cdot B_{12}N_{12/}\cdot \cdot \cdot {\mathrm{AlB}}_{11}N_{12}}$, M_CM_, and $M_{B_{12}N_{12}/{\mathrm{AlB}}_{11}N_{12}}$ introduce the *G*/*H*/*S* parameters of the optimized complexes, the CM drug, and B_12_N_12_/AlB_11_N_12_ nanocarriers, respectively. In addition, an insightful perspective on the desorption process was gained through calculating recovery time for all considered complexes, as illustrated in the following equation:

(20) $$t=v^{-1}exp\left(-\Delta G/KT\right)$$

where $\nu^{-1}$ addresses the attempt frequency of 10^–18^ s^−1^. K defines Boltzmann’s constant with a value of 0.00199 kcal/mol.K. T refers to temperature with a value of 310.15 K. All the employed DFT calculations were executed with the Gaussian 09 package [45] using the M06-2X method with the 6-311+G** basis set.

## 4. Conclusions

The adsorption process of the Chlormethine (CM) drug over the surfaces of the pure and Al-doped boron nitride (B_12_N_12_ and AlB_11_N_12_) nanocarriers was studied in-depth using various quantum mechanical calculations. From ESP analysis, the electrophilic and nucleophilic natures were affirmed for the studied nanocarriers and the drug, respectively. Moreover, the *V*_s,max_ value of Al over the surface of the Al-doped boron nitride (AlB_11_N_12_) nanocarrier was somewhat higher than B in the pure nanocarrier. The adsorption energy of the CM∙∙∙AlB_11_N_12_ complex within configuration A had the highest negative value, up to –52.40 kcal/mol. Upon SAPT results, electrostatic forces were deemed the predominant force, followed by induction. Furthermore, changes in electronic parameters and global indices of reactivity indicated the favorable contributions of the Al doping process to delivering CM. Delicately, thermodynamic parameters elucidated a spontaneous and exothermic nature for all considered complexes by negative Δ*G* and Δ*H* values, respectively. Negative solvation energy values highlighted the further preferentiality of the occurrence of the adsorption process in the water phase more than that in the gas phase. The overarching conclusions from the obtained results affirm the suitability of B_12_N_12_ and AlB_11_N_12_ nanocarriers in the adsorption process of CM, as well as the potent effect of the Al doping process on their features.

## Figures and Tables

**Table 1:** Interaction energy (*E*_int_, kcal/mol) of CM∙∙∙B_12_N_12_ and ∙∙∙AlB_11_N_12_ optimized complexes and adsorption energy (*E*_ads_, kcal/mol) within configurations A and B. Intermolecular distances (d) are given in Å.

Complex	Configuration	d	*E*_int_	*E*_ads_
CM∙∙∙B_12_N_12_	A	1.64	−59.55	–32.45
	B	2.82	−5.70	–5.15
CM∙∙∙AlB_11_N_12_	A	1.97	−59.68	–52.40
	B	2.32	−31.73	–28.15

**Table 2:** Total energy density (H_b_), Laplacian (∇^2^*ρ*_b_), electron density (*ρ*_b_), kinetic electron density (G_b_), local potential electron energy density (V_b_), and negative ratio of kinetic and potential electron energy density (−G_b_/V_b_) of the CM∙∙∙B_12_N_12_ and ∙∙∙AlB_11_N_12_ optimized complexes. All parameters are given in au.

Complex	Configuration	H_b_	∇^2^*ρ*_b_	*ρ*_b_	G_b_	V_b_	−G_b_/V_b_
CM∙∙∙B_12_N_12_	A	–0.0896	0.2414	0.1209	0.1499	–0.2395	0.6259
	B	0.0007	0.0387	0.0145	0.0090	–0.0083	1.0842
CM∙∙∙AlB_11_N_12_	A	–0.0046	0.3581	0.0655	0.0942	–0.0988	0.9530
	B	–0.0058	0.1633	0.0428	0.0466	–0.0524	0.8896

**Table 3:** The highest occupied molecular orbital (*E*_HOMO_), Fermi level (*E*_FL_), and the lowest unoccupied molecular orbital (*E*_LUMO_), as well as the energy gap (*E*_gap_) of optimized CM, B_12_N_12_, and AlB_11_N_12_ before and after adsorption process. All values are given in eV.

Drug/Nanocarrier/Complex	Configuration	*E*_HOMO_ (eV)	*E*_FL_ (eV)	*E*_LUMO_ (eV)	*E*_gap_ (eV)
CM		–8.28	–3.94	0.20	8.48
B_12_N_12_		–9.54	–5.12	–0.35	9.19
AlB_11_N_12_		–9.10	–7.05	–2.50	6.60
CM∙∙∙B_12_N_12_	A	–8.92	–4.93	–0.47	8.45
	B	–8.30	–4.51	–0.36	7.94
CM∙∙∙AlB_11_N_12_	A	–8.55	–4.96	–0.68	7.87
	B	–8.66	–5.12	–0.79	7.87

**Table 4:** Global indices of reactivity, including ionization potential (*IP*), electron affinity (*EA*), chemical potential (*μ*), global hardness (*η*), global softness (*S*), electrophilicity index (*ω*), and work function (*Φ*) calculated parameters for CM, B_12_N_12_, and AlB_11_N_12_ systems before and after adsorption.

Drug/Nanocarrier/Complex	Configuration	*IP* (eV)	*EA* (eV)	*μ* (eV)	*η* (eV)	*S* (eV^−1^)	*ω* (eV)	*Φ* (eV)
CM		8.28	–0.20	–4.04	4.24	0.24	1.92	3.94
B_12_N_12_		9.54	0.35	–4.94	4.60	0.22	2.66	5.12
AlB_11_N_12_		9.10	2.50	–5.80	3.30	0.30	5.10	7.05
CM∙∙∙B_12_N_12_	A	8.92	0.47	–4.70	4.22	0.24	2.61	4.93
	B	8.30	0.36	–4.33	3.97	0.25	2.36	4.51
CM∙∙∙ AlB_11_N_12_	A	8.55	0.68	–4.62	3.93	0.25	2.71	4.96
	B	8.66	0.79	–4.72	3.93	0.25	2.84	5.12

**Table 5:** Adsorption ($E_{\mathrm{ads}}^{\mathrm{solvent}}$ ) and solvation (Δ*E*_solv_) energies for the CM∙∙∙B_12_N_12_ and ∙∙∙AlB_11_N_12_ optimized complexes in the water phase. All energies are calculated in kcal/mol.

Complex	Configuration	$E_{\mathrm{ads}}^{\mathrm{solvent}}\;(\mathrm{kcal}/\mathrm{mol})$	Δ*E*_solv_ ^a^ (kcal/mol)
CM∙∙∙B_12_N_12_	A	–34.22	–10.52
	B	–4.06	–7.65
CM∙∙∙AlB_11_N_12_	A	–53.50	–15.81
	B	–27.17	–13.72

^a^ $\Delta E_{\mathrm{solv}}=E_{\mathrm{solvent}}-E_{gas}$.

**Table 6:** Thermodynamic parameters, i.e., Gibbs free energy (Δ*G*), enthalpy change (Δ*H*), and entropy change (Δ*S*), of the CM∙∙∙B_12_N_12_ and ∙∙∙AlB_11_N_12_ complexes computed in kcal/mol.

Complex	Configuration	Δ*G*	Δ*H*	Δ*S*
CM∙∙∙B_12_N_12_	A	–14.72	–29.94	–0.05
	B	–5.77	–3.97	–0.03
CM∙∙∙AlB_11_N_12_	A	–37.15	–50.14	–0.04
	B	–15.71	–26.79	–0.04

## Data Availability

Data is contained within the article.
